# Curcumin Ameliorates Aluminum Nanoparticle‐Induced Cognitive Impairment by Enhancing BDNF‐Akt Signaling in the Mouse Hippocampus

**DOI:** 10.1002/alz70855_097131

**Published:** 2025-12-23

**Authors:** Maryam Moosavi, Samaneh Reiszadeh Jahromi

**Affiliations:** ^1^ Nanomedicine and Nanobiology Research Center, Shiraz, Fars, Iran (Islamic Republic of); ^2^ University of Sistan and Balouchestan, Zahedan, Zahedan, Iran (Islamic Republic of)

## Abstract

**Background:**

Aluminum (Al) exposure has been linked to Alzheimer's Disease (AD) pathogenesis. Al nanoparticles (AlNPs) exhibit significantly higher neurotoxicity compared to Al ions. Curcumin, a natural compound with neuroprotective properties, was investigated for its ability to mitigate AlNP‐induced cognitive impairment.

**Method:**

Adult male Swiss mice were administered AlNPs (10 mg/kg, subcutaneous) alone or in combination with curcumin (2.5 or 25 mg/kg, oral) for 10 days. Cognitive function was assessed using the passive avoidance test, while anxiety‐like behavior was evaluated using the elevated plus maze. Western blot analysis was employed to evaluate the expression levels of BDNF and phosphorylated Akt in the hippocampus.

**Result:**

Results demonstrated that AlNP exposure significantly impaired cognitive function in mice, while curcumin treatment, particularly at 25 mg/kg, significantly ameliorated this impairment. AlNP treatment significantly decreased hippocampal BDNF and phosphorylated Akt levels, indicating a downregulation of the BDNF‐Akt signaling pathway. Notably, curcumin treatment significantly increased BDNF and phosphorylated Akt levels compared to the AlNP‐treated group, suggesting an enhancement of this neuroprotective pathway.

**Conclusion:**

These findings suggest that curcumin exerts neuroprotective effects against AlNP‐induced cognitive impairment, potentially through the enhancement of BDNF‐Akt signaling in the hippocampus. These results highlight the potential therapeutic implications of curcumin as a natural intervention for mitigating the neurotoxic effects of AlNPs.

